# Advancements and challenges in bloodstain pattern analysis: Addressing limitations for enhanced forensic reliability

**DOI:** 10.1007/s00414-026-03817-x

**Published:** 2026-04-27

**Authors:** Mayur Sudhir Balbudhe, B. Suresh Kumar Shetty, Riya Render, Nayanatara Arun Kumar

**Affiliations:** 1 Department of Forensic Medicine and Toxicology, Kasturba Medical College Mangalore, Manipal Academy of Higher Education, Manipal, India; 2 Department of Physiology, Kasturba Medical College Mangalore, Manipal Academy of Higher Education, Manipal, India

**Keywords:** Bloodstain pattern analysis, Forensic limitations, Error rates, Cognitive bias, Surface interactions, Crime scene reconstruction, Reliability enhancement

## Abstract

Bloodstain Pattern Analysis (BPA) is a technique used in forensic investigations to recreate violent crime scenes by examining the distribution of bloodstain patterns to interpret the dynamics of the occurrence, including the impact directions, movement of the participants, and the type of weapon that may have been used. Its interpretiveness is, however, severely limited due to underlying limitations, such as the ambiguity of theories of causation patterns, large classification error rates (e.g., 23–30% in controlled studies), distortions created by the surface, environmental contaminations, and a tendency to cognitive errors. This comprehensive survey critically examines these issues, drawing on current meta-analyses and empirical evidence to identify the shortcomings of one-to-many mappings, where a network of mechanisms generates identical patterns, and how these issues can be used to assess judicial reliability. Such influential factors include the biological (e.g. viscosity changes), and physical aspects (e.g. surface tension) of blood, and the surface textures, impact velocities (classified as low: 5 ft/s or less; medium: 5–25 ft/s, high: >100 ft/s), and the fabric properties such as weave density and the rate of absorption, which can change the morphology of the stain by up to 40%. The error rates in fabric classifications are found to be 23.4%, and satellite stains are misidentified at a rate of 59%, which is exacerbated by false contextual transformations. In response to this, the review suggests refined element-based classification systems (e.g., OSAC 2022-S-0030), bias-reduction measures such as Linear Sequential Unmasking Expanded (LSU-E), and technological integrations, including 3D laser scanning and machine-learning-based automated feature extraction. Future research perspectives, such as interlaboratory validation and regional surface data, are suggested to promote the scientific rigour of BPA, especially when using porous textiles, which are dominant in various parts of Asia. This objective analysis highlights the expediency of BPA, though caution is needed to avoid the phenomenon of overreaching evidence.

## Introduction

Forensic science combines physics, biology, and chemistry to reconstruct criminal activities and support legal arguments [1–3]. Bloodstain Pattern Analysis (BPA), which is one of the pillars of violent crime investigations, analyses the shape, size, distribution, and location of bloodstains to infer mechanistic information, such as the sequence of attacks, the relative positions of the victim and perpetrator, and the instrumentalities, e.g. blunt force or projectiles [4, 5]. Based on the exchange principle of Locard, which states that every physical contact leaves an imprint in a trace, BPA utilises stain patterns and morphologies to either dismiss or corroborate alibis, chronology, and stories [6, 7]. It has historical origins in the directional inference of head-trauma spatter work of Eduard Piotrowski in 1895, in his dissertation, which determined parabolic trajectories [3, 7], developed by Herbert MacDonell in the 1970s, in his seminal works, and the 1983 formation of the Bloodstain Pattern Analysis Subcommittee of the International Association to Study Identification [8].

However, the alleged accuracy of BPA is a sham. Even a 2025 overview acknowledges its fragile position: theoretically based on fluid dynamics, but in practice, ambiguities of causation, such as one-to-many, are found, with various mechanisms (such as arterial spurts versus cast-off) simulating its results [9]. Reliability is affected by inconsistencies between different analysts (a best agreement of 60–70%), 30% error rates during blind tests, and cognitive biases that can falsely identify people [9–11]. These drawbacks have been identified in BPA cases, as demonstrated by reports prepared by the U.S. National Academy of Sciences (2009) and the President’s Council of Advisors on Science and Technology (2016), which criticise the forensic disciplines due to the absence of empirical grounds [9].

The review avoids the crude expositions of BPA, such as the categorisation of velocities or the definition of passive stain, which are typically introductory to textbook treatments [4, 5], to explore its subject weaknesses and salvage strategies. It measures limitations by combining 2025 insights [9], categorises surface confounds by using comparative data, and plots progress by bias-proofing and automation. Local validation is essential in India and Asia, where drying distortions rise faster than in other regions, because mannerisms to absorb errors are higher (i.e. cotton saris), which make such errors more severe [12, 13]. Finally, the rebranding of BPA as an aiding, probabilistic instrument, as opposed to a deterministic oracle, makes the product more legitimate within the field of forensics.

### Weaknesses of bloodstain pattern analysis

The interpretive scaffold of BPA, which combines empirical heuristics and physics-grounded models, is marred by a myriad of drawbacks, including theoretical, empirical, environmental, and human weaknesses. These undermine its ability to make unequivocal reconstructions because patterns can tend not to reverse-engineer because of multifactorial noise [9].

### Theoretical and validity problems

The validity of BPA is based on deterministic relationships between deposition processes and observable behaviours, as determined by the Navier-Stokes equations of droplet impact and the Rayleigh-Plateau instability of spatter evolution [5, 9]. These are theoretically sound and exhibit predictable morphologies in idealised conditions (e.g., a spherical drop at 90° incidence). However, in practice, the validity breaks down in the case of one-to-many mappings: a fine spray (less than 1 mm) may result from gunshot residue, high-velocity coughing, or aerosolised cast-off, leading to cross-gunned attributions [5, 9, 12]. The best that impact velocity thresholds (low: gravity-driven drips 4 + mm diameter), (medium): blunt trauma, 1–4 mm), and (high): projectiles, < 1 mm) can be probabilistic due to their lack of consideration of air resistance, droplet coalescence, non-Newtonian rheology of blood (viscosity increasing 10–20% after coagulation) [9, 14, 15].

The parabolic arcs of Piotrowski, for example, assume the same ejection velocity (5–10 m/s in the case of arterial), but empirical measurements show a 20–30% error on non-horizontal surfaces [3, 7, 9]. Without detailed cause-and-effect databases, BPA loses its predictive power. By 2025, an analysis decries the lack of peer-reviewed literature connecting mechanisms and patterns, noting that only correlating validity estimates of less than 0.6 have been achieved in simulation [9]. Such a theoretical gap is reflected in the exaggerations of courts, where unproven inferences (e.g., defensive wounds caused by cast-off) can be challenged by Daubert [9].

### Errors of classification and reproducibility

The tripartite taxonomy (passive, e.g., drips, pools), spatter (e.g., impact, projection), and altered (e.g., diluted, clotted) used by BPA in its reconstructions is found to be a valid conceptual framework. However, it is ineffective in practice, with feature discernment relying on the subjective interpretation of features [4, 9]. Inter-rater agreement is at 60–70% on hard surfaces, but on textiles, the inter-rater agreement is lower at 50%, as determined by blinded experiments [9, 10].

Table 1 lists cornerstone studies, in which gradients of errors are found: the two papers of Taylor et al. (2016) measured 23.4% overall misclassifications on textiles, the drip satellites (59% error) and expirations (16% error) were most susceptible, since wicking as an effect of weave hides edges [10, 11]. Hicklin et al. (2021) generalised this to holistic findings, with only 65 per cent reproducibility on composite scenes among 50 analysts [9]. Similar results in fingerprint variances (Ulery et al., 2012) highlight the ubiquity of forensic science, and the error of BPA increased due to pattern ambiguity [9]. It is exacerbated by methodological heterogeneity, e.g., different magnification or lighting; novices can make errors 25% more frequently than veterans on ambiguous substrates [9, 11].

**Table 1:** Key studies on BPA classification reliability and error rates

Study	Year	Focus	Key Finding	Error/Reproducibility Rate
Taylor et al. (Part 1)	2016	Rigid non-absorbent surfaces	Variability in impact vs. cast-off distinction; subjective edge criteria.	20–25% misclassification
Taylor et al. (Part 2)	2016	Fabric surfaces	Satellite stains from drips are most misidentified; the extent of the stain aids accuracy.	23.4% overall; 59% for satellites
Osborne et al.	(2016)	Contextual effects on fabrics	Negative cues inflate errors; pattern size inversely correlates.	15% bias-induced drop
Hicklin et al.	(2021)	Holistic scene conclusions	Inconsistent origin estimations across analysts.	35% inter-rater variance
[23] (Meta-review)	2025	Aggregate reproducibility	One-to-many mappings underlie 10–30% blind-test errors.	< 70% agreement typical

### Surface and environmental confounders

The friction of surfaces determines the stain fidelity, rather than the fall height, and texture disrupts cohesion through fragmentation caused by asperity [9, 16, 17]. Nonporous smooths (glass: low splatter, circular stains) are the opposite of porous roughs (wood: high satellites, 30–50% diameter reduction), and pig-blood trials (3–4.3 mm droplets, 2.4–4.9 m/s) [15].

Equations like $\:D=k\sqrt{V\cdot\:d/R}$ (stain diameter * D*, velocity * V*, droplet size * d*, roughness * R*) approximate but falter on fabrics, where capillary imbibition swells stains 20–40% [13, 15, 18].

The differences in morphologies among archetypes are discussed in Table 2, which incorporates species variances (human ≈ pig; goat/chicken spurrier) [8, 19, 20]. Heat (24–250 °C) induces boiling (nucleation > film), resulting in a shrinkage of 15%. Humidity inhibits drying, and ageing causes skewing of hours [15, 21, 22]. Arson soot conceals pre-incendiary prints, and laundering removes evidence from textiles 25 per cent [9, 10, 13].

**Table 2:** Comparative effects of surface types on bloodstain morphology (Based on Human/Pig Blood Simulations)

Surface Type	Texture/Porosity	Typical Stain Features	Splatter Volume	Distortion Factor	Example Implications
Smooth Non-Porous (e.g., Glass/Tile)	Low/Non	Circular, well-defined edges; spines are rare	Low (< 10% fragmentation)	Minimal (5–10%)	Accurate velocity estimates; low error
Rough Non-Porous (e.g., Sandblasted Steel)	High/Non	Irregular edges, merged spines; satellites	Medium (20–30%)	Moderate (15–25%)	Overestimated height; 20% misclass.
Porous Hard (e.g., Raw Wood/Asbestos)	Medium/High	Absorbed cores, diffused halos	Low-Medium (10–25%)	High (20–35%)	Pool penetration; fabric-like wicking
Soft Absorbent (e.g., Paper Towel/Textile)	High/High	Spread pools, fibre-clinging droplets	High (> 40%)	Severe (30–50%)	59% satellite errors; bias-prone

### Cognitive biases and human factors

The craftsmanship of BPA is an open door to prejudices, in which irrelevant information (e.g., suspect motives) distorts perceptions through confirmation biases [9]. According to Cooper et al. (2019), 50 or more experiments of forensic bias are identified in the meta-review, and BPA analogues exhibit swings in accuracy of 10–20% [9]. Quantification of contextual sway was conducted by Osborne et al. (2016), who found that affirming narratives reduced errors by 12% and misleading ones by 18% [9, 11]. Kassin et al. (2013) position this as a case of tunnel vision, which is exacerbated by analyst fatigue or caseloads [9]. Dror (2023) recommends LSU-E, which stages the information to create quarantine biases, aiming to achieve 25% reproducibility gains in pilots [9] (Table 3).

**Table 3:** Cognitive biases impacting BPA interpretations

Bias Type	Mechanism	BPA Manifestation	Mitigation (per [23])	Error Inflation
Confirmation	Seeking pattern-congruent evidence	Overfitting spatters to “gunshot” narrative	LSU-E sequencing	10–15%
Contextual	External cues override stains	Misclassifying drips as expired per the witness	Blind analysis protocols	15–20%
Expectation	Preconceived scene dynamics	Ignoring surface effects in reconstructions	Bias-awareness training	5–10%
Anchoring	Initial impressions dominating	Fixating on velocity thresholds	Element-based classification	8–12%

### Precision: Technological integrations

Volumetric distributions are measured using 3D scanning (e.g., FARO Focus) and VR simulations; they reduce by 15–20% errors at the origin of slashing, through ray-tracing [9, 20, 23]. Map360 combines scans using ML classifiers (e.g., SHAP to estimate the number of drips), and 85 per cent of drip detection is automated compared to 70 per cent detected manually [9]. Near-infrared spectroscopy can penetrate through fabric, recovering up to 30 per cent of hidden stains [9, 13].

### Refined methodologies and bias countermeasures

OSAC’s 2022-S-0030 focuses on granular elements (e.g., edge irregularity metrics), which boosts agreement by 10–15% [9]. LSU-E (Dror et al., 2021) enforces unmasked tiers, curbing contextual noise [9]. IABPA’s bias modules simulate vignettes, mandating audits for certification [8, 9] (Table 4).

**Table 4:** Advancements and Their Mitigation of BPA Limitations

Advancement	Description	Targeted Limitation(s)	Impact/Evidence (per [23])
3D Laser Scanning/VR	Geometric capture for trajectory modelling	Validity, Reproducibility	15–20% error reduction in origins
ML Feature Extraction	Automated spine/satellite detection via neural nets	Classification Errors	85% accuracy on ambiguous patterns
OSAC Element Standards	Measurable traits over holistic judgments	Theoretical, Reliability	10–15% inter-rater improvement
LSU-E Protocols	Phased info release to isolate biases	Cognitive Biases	25% reproducibility gain in trials
Interlab Datasets	Standardised simulations for error benchmarking	Error Rates, Surface Confounds	Targets < 10% aggregate errors

### Future research areas of enhancement

Focus on cause-pattern validations through high-fidelity sims (e.g. CFD modelling) [9]. The errors of Asian fabrics had been reduced by half by fabric consortia — cataloguing weaves/absorption [9, 12, 13]. The longitudinal experiments on VR admissibility and species-neutral baselines (human-centric) are encouraged [8, 9, 19]. By utilising collaborative tools such as the IABPA repository, meta-analyses are encouraged with the goal of achieving error rates of less than 10 per cent by 2030 [9].

## Conclusion

The forensic potential of BPA disentangling violence, through stains in a controversial manner, is hedged with significant limitations: a lack of validity due to the causal multiplicities, the lack of reliability due to 23–35% error rates, the confounds of surfaces/environment, and a bias of 10–20 per cent of calls [9–11]. These are tabulated herein, and they bring about a paradigm shift from certitude to calibrated probability, which prevents miscarriages in various locales, such as India, where textiles and monsoons compound distortions [12]. The innovations, 3D/ML accuracy, and OSAC/LSU-E protection are signs of reliability, but require strict scrutiny [9]. Investing in education, datasets, and humility will transform BPA into a trusted partner of justice, reconciling intuition with empirical evidence.
